# Parasympathetic Tone Selectively Modulates Electrogram Fragmentation and Spectral Properties in Right Atrial Ganglionated Plexi

**DOI:** 10.1002/joa3.70424

**Published:** 2026-07-29

**Authors:** Andrea Giomi, Andrea Bernardini, Alessandro Paoletti Perini, Margherita Padeletti, Cristiano Salvatore Zaccaria, Federica Michelotti, Umberto Signorini, Rossella Marcucci, Massimo Milli

**Affiliations:** ^1^ Department of Medical Specialities Azienda USL Toscana Centro, Santa Maria Nuova Hospital Florence Italy; ^2^ Department of Experimental and Clinical Medicine University of Florence Florence Italy

**Keywords:** autonomic tone, cardioneuromodulation, EGM fragmentation, ganglionated plexi, spectroscopy

## Abstract

**Background and Aims:**

EGM fragmentation and spectral analysis have both been proposed to identify ganglionated plexi (GP) regions in the context of cardioneuroablation. However, their correlation has never been fully investigated, and it remains unclear whether these properties are related to structural or functional characteristics. The aim of this study is to evaluate how these features are related and modulated by parasympathetic tone.

**Methods:**

The study population included 13 patients (mean age 34 ± 12 years, 69% male) undergoing an electrophysiological study. High‐density right atrial mapping was performed using a multielectrode catheter during baseline conditions and after atropine administration. Spectral analysis of each point was obtained by fast Fourier transformation, deriving mean, median, and skewness.

**Results:**

Baseline maps showed fragmented EGM areas in the posterior‐septal wall, corresponding to right atrial GP distribution. When compared to the remaining atrial myocardium, GP regions showed greater mean and median frequencies and lower skewness (*p* < 0.001). Following atropine, only GP regions showed a significant decrease in mean (112.6 ± 13.9 to 101 ± 8.1 Hz, *p* = 0.02) and median frequency (*p* = 0.02), as well as an increase in skewness (0.03 ± 0.43 to 0.30 ± 0.27, *p* = 0.015), indicating a shift toward the spectral profile of compact myocardium. In non‐GP locations, no significant spectral alterations were found.

**Conclusions:**

EGM fragmentation and spectral properties are strongly correlated. Parasympathetic tone modulates both features exclusively in GP areas. The distinct signal pattern of GP regions may be a result of both local cholinergic release and structural cellular discontinuity with potential implications for a more precise targeting in the context of CNA.

## Graphical abstract

Electrogram fragmentation and spectral EGMs features in GPs area are selectively modulated by parasympathetic tone, as demonstrated by high‐density right atrial mapping before and after vagal blockade. EGMs, bipolar atrial electrograms; GPs, ganglionated plexi; RA, right atrium.

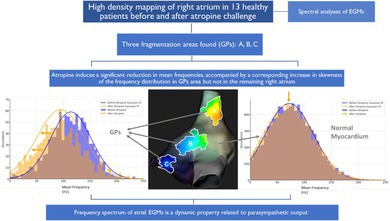

**Abbreviations**
ACh: acetylcholine
AF: atrial fibrillation
BMI: body mass index
CNA: cardioneuroablation
EAM: electroanatomical mapping
ECG: electrocardiogram
EGM: bipolar atrial electrogram
EPS: electrophysiological study
FEGM: fractionated electrogram
FFT: fast fourier transform
GP: ganglionated plexus
GPA: ganglionated plexi ablation
HFS: high‐frequency stimulation
LVEF: left ventricular ejection fraction
M: mean
MAP‐0: baseline map acquired during sinus bradycardia
MAP‐ATP: map acquired after atropine administration
NMS: neuro‐mediated syncope
RA: right atrium
RF: radiofrequency
SA: spectral analysis
SD: standard deviation
SVT: supraventricular tachycardia

## 1 Introduction

Ganglionated plexi (GPs) are nervous stations located in the epicardial fat pads on the external surface of the heart. They are composed of a heterogeneous population of neurons, including sympathetic and parasympathetic elements as well as interconnecting neurons and play an important role in the autonomic regulation of cardiac rhythm, receiving input from the higher nervous centers and sending output to nearby myocardial tissue, thus controlling the activity of the sinoatrial and atrioventricular nodes, as the working myocardium [1, 2, 3, 4]. The role of GPs ablation (GPA) for antiarrhythmic purposes was first recognized in the setting of atrial fibrillation (AF) where this approach showed additive efficacy in patients with “vagal AF” [5, 6]. More recently GPA has expanded as an alternative to pacemaker implantation in the treatment of neuro‐mediated syncope (NMS) [7, 8, 9, 10, 11, 12]. However, a gold standard for localizing GPs during electrophysiological procedures has not yet been established. Different methods have been proposed and each one has its own limitations [13]. Fragmentation of endocardial bipolar electrograms (EGMs) is a well‐established surrogate of local innervation and represents one of the simplest and most available marker of GPs areas. Spectral analysis (SA) represented the pioneering methodology for the identification of atrial innervation sites [14]. However, its subsequent clinical adoption has been limited due to the high complexity of data processing and the restricted integration capabilities within modern electroanatomical mapping (EAM) platforms. However, the actual correlation between Spectral analysis and EGM fragmentation has never been systematically evaluated, and it remains unclear whether these properties are related to structural or functional characteristics. The aim of this study is to investigate the correlation between EGMs fragmentation and their spectral characteristics and to evaluate these properties in relation to vagal tone.

## 2 Methods

### 2.1 Patient's Selection

Thirteen patients who were referred to our Center for an electrophysiological study (EPS) were prospectively recruited. Indication for EPS included a previously documented supraventricular tachycardia (SVT), ventricular pre‐excitation, recurrent palpitations, syncope of unknown origin. Exclusion criteria were: age < 18 or > 50 years, structural heart disease, history of atrial fibrillation, previous ablation, systemic or metabolic diseases. Moreover, patients with a history of glaucoma, myasthenia gravis, pyloric stenosis, thyrotoxicosis, allergy to atropine, known obstructive gastrointestinal or urinary disease were considered at risk of side effects from atropine and were not included in this study. Any antiarrhythmic drugs or those with possibly neuromodulatory properties were discontinued for at least five half‐lives before the procedure. All patients underwent a careful cardiovascular examination and a transthoracic echocardiography. This study represents a complementary analysis of a prospectively enrolled cohort previously reported by our group [15] in which the spatial distribution of EGM fragmentation was characterized. The present analysis specifically focuses on the spectral properties of atrial electrograms, which were not addressed in the prior work. The study complies with the Declaration of Helsinki, was approved by the local Ethical Committee, and all participants provided written informed consent.

### 2.2 Patient Preparation and Basal EPS

Patients were monitored with 12‐lead ECG, noninvasive blood pressure measurements, and pulse oximetry during the entire procedure. After local groin anesthesia with 400 mg subcutaneous lidocaine infusion, bilateral femoral vein accesses were obtained. Two or more multipolar catheters were placed in the right chambers according to the scheduled procedure. Baseline EPS was performed according to the patient's indication until the diagnostic workflow was completed. A radiofrequency ablation of arrhythmic substrate was carried out when indicated, in accordance with current guidelines [16]. When ablation was required for right‐sided substrates (such as the slow pathway or right accessory pathways), it was performed only after the mapping phase to avoid including ablation lesions in the maps.

### 2.3 High Density Mapping

An electroanatomic three‐dimensional high‐density mapping of the right atrium was performed by Rhythmia HDx (Boston Scientific, Marlborough, MA) using a multipolar mapping catheter IntellaMap Orion (Boston Scientific, Marlborough, MA). EGMs were acquired including low and high frequency components (0.5–500 Hz bandpass filter). Baseline map (MAP‐0) was acquired during sinus bradycardia in order to maximize the vagal effect on atrial EGMs. In patients with a resting heart rate above 80 bpm or with an evident state of anxiety, mild sedation with intravenous benzodiazepine was performed. A minimum of 5000 points were collected with a uniform distribution throughout the anatomy of the right atrium, including the proximal borders of both the superior and inferior vena cava. A 2 mg bolus of atropine was then administered, and a second map (MAP‐ATP) was acquired with the same methods, only once a clear vagolytic effect was achieved, as evidenced by an increase in heart rate of at least 25% [17].

### 2.4 Data and Spectral Analysis

**FIGURE 1:**
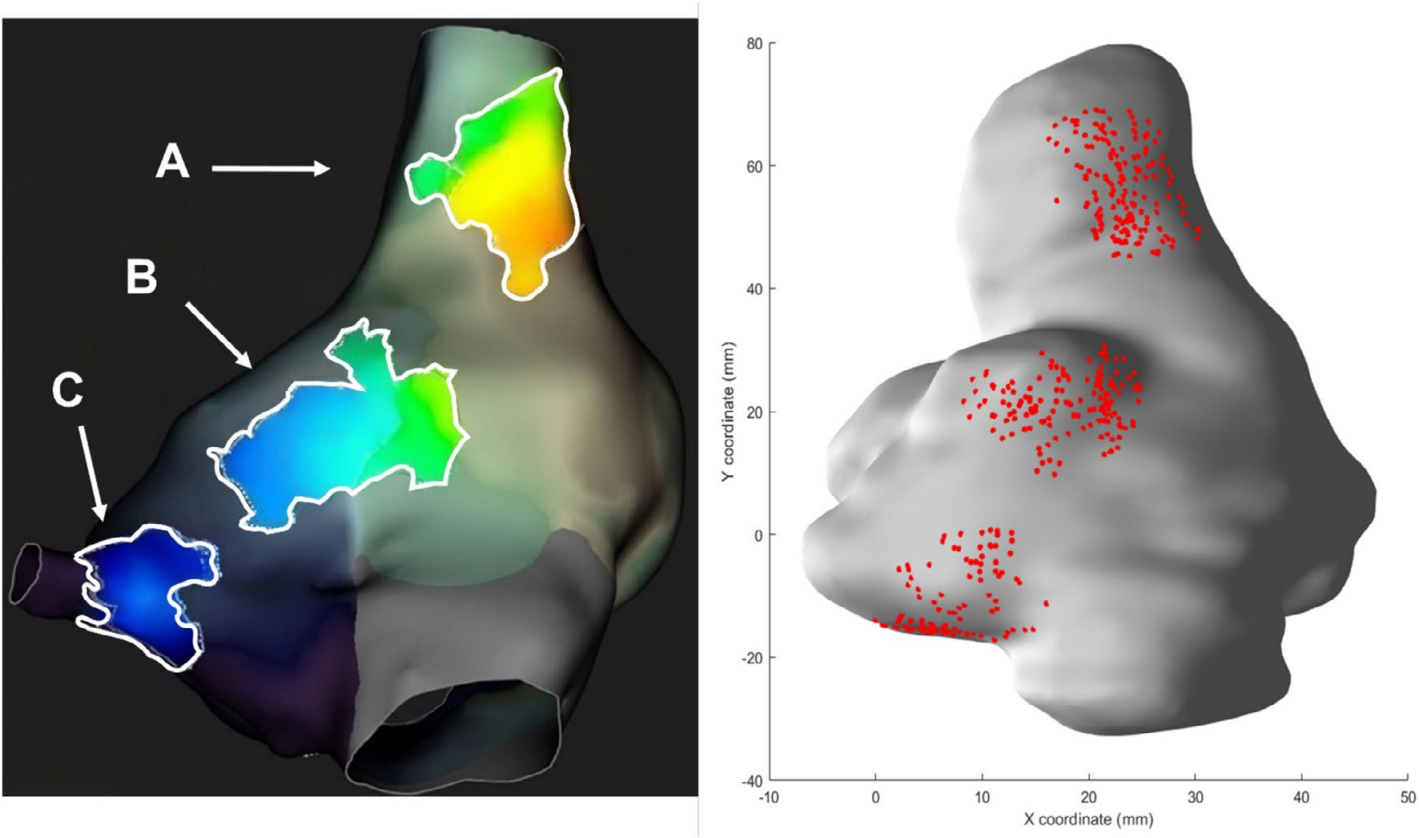
Left side: Localization of atrial fractionated electrograms in the right atrium (electroanatomic map). Three main areas were identified in the supero‐, mid‐, and infero‐position, referred to as A, B, C, corresponding to: *Superior‐paraseptal* (A), *posterior‐right atrial* (B) and *inferior‐paraseptal* (C) GPs. Right side: Mathlab reconstruction of the corresponding electroanatomic map to analyze spectral distribution of EGMs, computing the frequency spectrum of each point using Fast Fourier Transformation (FFT). Red dots correspond to fragmented electrograms areas, while the remaining right atrial myocardium (gray area), was analyzed separately.

An offline analysis was conducted to compare MAP‐0 and MAP‐ATP for each patient, starting from the localization of atrial fractionated electrograms (FEGMs). Fragmentation was defined as the presence of four or more deflections in the local bipolar signal, a threshold previously reported as the best predictor of parasympathetic response [18]. FEGM areas were highlighted and measured using the Lumipoint module (Boston Scientific, Marlborough, MA). Different degrees of fragmentation were analyzed for each map by adjusting the Lumipoint threshold to more than 4 deflections (Figure 1, left side). Afterwards, our evaluation focused on the spectral analysis of electrograms (EGMs), computing the frequency spectrum of each point using Fast Fourier Transformation (FFT) in Matlab (MathWorks, version R2024b; Figure 1, right side). Signals were acquired at a sampling frequency of 953.7 Hz. As exported, the electrograms had already been processed by the mapping system with a 0.5–500 Hz band‐pass filter and a 50 Hz notch filter to remove baseline wander, high‐frequency noise, and power‐line interference; no further filtering was applied in MATLAB, so that the full frequency content of the signal was preserved for analysis. Before computing the spectrum, the mean value of each electrogram was subtracted to remove the DC component. For each mapping point, the analyzed signal corresponded to the entire bipolar electrogram contained within the local mapping window. The mapping window was set to begin approximately 30 ms before P‐wave onset and to extend approximately 50 ms beyond P‐wave termination; accordingly, each electrogram comprised between 150 and 240 samples, corresponding to a time window of 158–252 ms. The variability in window duration across patients reflected differences in intra‐atrial conduction delay and P‐wave duration. A rectangular window equal to the full length of the electrogram was used, since shortening the analysis window would have reduced both the spectral resolution and the ability to detect shifts in the frequency distribution. The power spectral density (PSD) was estimated through the periodogram, computed as the squared modulus of the fast Fourier transform. Frequency distribution was then analyzed separately for fragmented regions (≥ 4 deflections) and the remaining right atrial myocardium (whole right atrium excluding the fragmented area). From the PSD, the mean and median frequency were derived (MATLAB functions *meanfreq* and *medfreq*) and used as the metrics to quantify the spectral shift before and after atropine administration, both in GP regions and in the remaining atrial myocardium. The median frequency was retained as a complementary descriptor because the mean frequency may remain unchanged between two distributions whose spectral dispersion differs. From the software analysis, we obtained also parameters of distribution as kurtosis, and skewness. Skewness, a measure of the asymmetry of a distribution, was particularly noted: an increase in skewness was interpreted as a leftward shift in the distribution graph, indicating a predominance of lower values of frequencies.

### 2.5 Statistical Analysis

Continuous variables were expressed as mean (M) and standard deviation (SD), while categorical variables as number (*n*) and percentage (%). Normal distribution of data was assessed by the Shapiro–Wilk test. For continuous variables and independent samples, comparison between groups was evaluated using the unpaired *t*‐test for normally distributed variables or Mann–Whitney *U* test for non‐normally distributed data. Pearson *χ* ^2^ test was applied for categorical variables. In case of paired samples, for continuous variables, comparison between groups was evaluated using paired *t*‐test for normally distributed variables or Wilcoxon signed rank test for non‐normally distributed data, while McNamar test was applied for categorical variables. A *p*‐value < 0.05 was considered statistically significant. Statistical analysis was performed with SPSS Statistics version 22 (IBM Corporation).

## 3 Results

### 3.1 Patient Population and EPS

The study population included 13 patients with a mean age of 34 ± 12 years, of whom 9 were male (69.2%). In 11 patients (84%), the indication for EPS was supraventricular tachycardia. The remaining 2 patients were referred for palpitations and syncope of unknown origin, respectively (Table 1).

**TABLE 1:** Baseline and procedural characteristics of the study population.

Variables	All patient (*N* = 13)
Age, years	33.9 ± 12.4
Male, *n* (%)	9 (69.2%)
BMI, kg/m^2^	23.9 ± 2.6
Hypertension, *n* (%)	1 (7.7%)
Dyslipidemia, *n* (%)	1 (7.7%)
Smokers, *n* (%)	4 (30.8%)
Structural cardiomyopathy *n* (%)	0 (0)
LVEF (%)	64 ± 5
Basal heart rate during mapping (bpm)	69 ± 7
Indications for EPS	
SVT, *n* (%)	12 (92.3%)
Syncope, *n* (%)	1 (7.7%)
Medications	
Ramipril, *n* (%)	1 (7.7%)
Propafenon, *n* (%)	1 (7.7%)
Flecainide, *n* (%)	1 (7.7%)

*Note:* Data are presented as: Mean ± standard deviation or *n* (percentage).

Abbreviations: BMI, body mass index; LVEF, left ventricular ejection fraction; SVT, supraventricular tachycardia.

### 3.2 High‐Density Mapping and Spectral Analysis

MAP‐0 and MAP‐ATP were both acquired in all patient during sinus rhythm without significant differences in density of points (8521.7 ± 1517.1 vs. 8512.5 ± 1085.5, *p* = 0.97). The response to atropine was documented by a mean heart rate increase of 59% ± 23% from baseline. All basal maps showed a similar pattern of fragmentation in the posterior‐septal wall reflecting the anatomical distribution of right GPs. In particular, three main areas were identified in the supero‐, mid‐, and infero‐position, referred to as A, B, C, corresponding to: *superior‐paraseptal* (A), *posterior‐right atrial* (B) and *inferior‐paraseptal* (C) GPs, according to the latest standardized nomenclature [17] (Figure 1).

**FIGURE 2:**
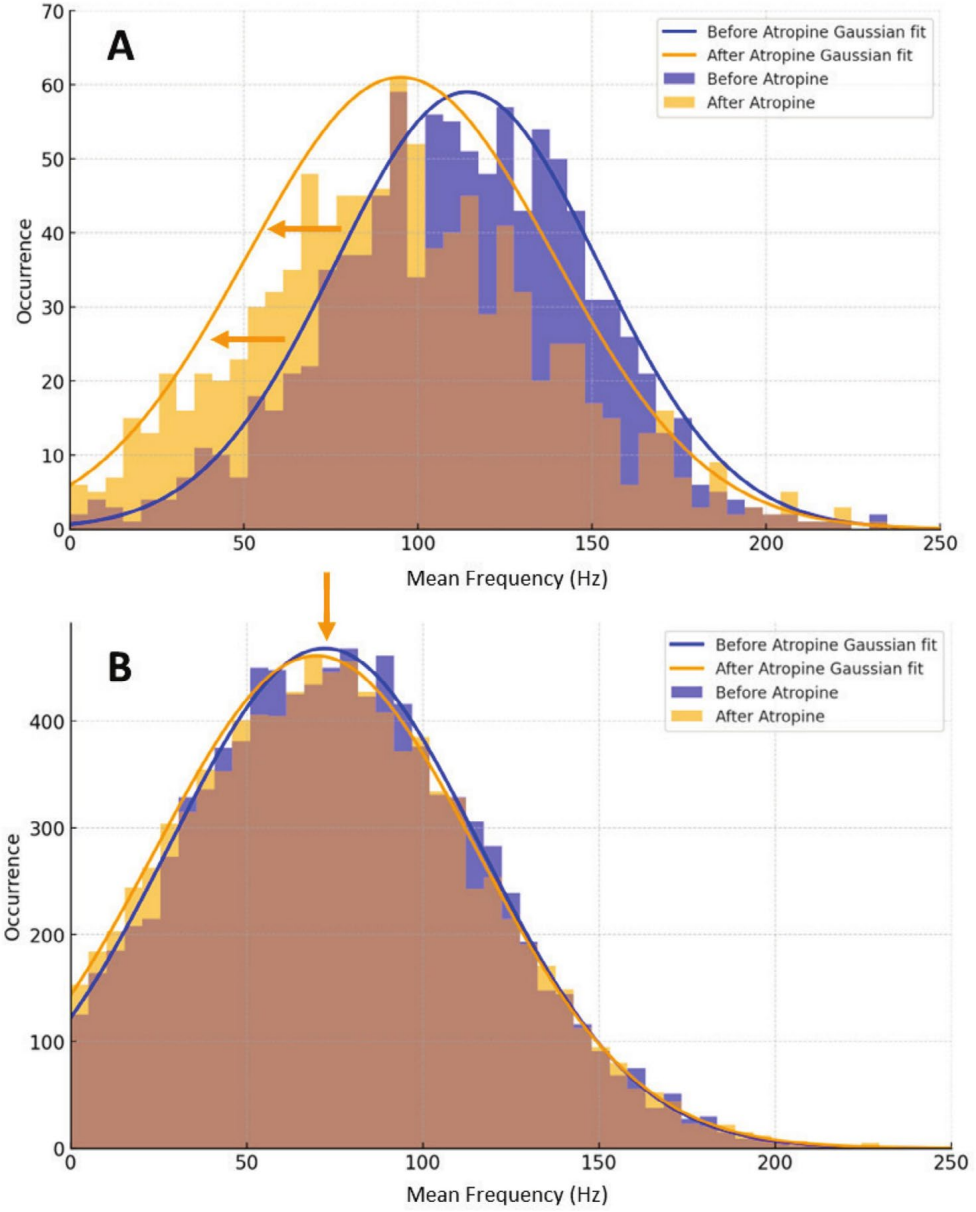
Spectral analysis and atropine changes. Panel A: Distribution of mean frequencies in GPs. Baseline frequency distribution is characterized by a higher mean frequency and a lower skewness in comparison with the remaining atrial myocardium. Following the atropine challenge, a significant reduction in mean frequencies was observed, accompanied by a corresponding increase in skewness of the frequency distribution (yellow line and arrows). Panel B: In the remaining atrial myocardium, atropine infusion had no effect on mean frequency and did not induce a shift in skewness of distribution. Brown areas represent regions of overlap between the spectral analysis before and after atropine infusion. The figure was realized on a single patient, taken as a reference.

GPs A, B, and C exhibited a distinct baseline frequency distribution compared to the rest of the right atrial myocardium, characterized by a higher mean and median frequency and a lower skewness (*p* < 0.001 for all; Table 2). Following the atropine challenge, a significant reduction in mean and median frequency was observed only in GPs regions A, B, and C (from 112.6 ± 13.9 Hz to 101 ± 8.1 Hz, *p* = 0.02, and from 113.4 ± 18.9 Hz to 98.4 ± 16.8 Hz, *p* = 0.02, respectively). Notably, despite this reduction, mean and median frequencies in GP regions after vagolysis remained significantly higher than those of the surrounding atrial myocardium. The reduction in frequency was accompanied by a corresponding increase in skewness of the frequency distribution (from 0.03 ± 0.43 to 0.3 ± 0.27, *p* = 0.015), indicating a shift toward lower frequencies in the spectral distribution (Table 2; Figure 2A). In contrast, atropine administration had no effect on mean or median frequency distribution in the remaining atrial myocardium (from 85.6 ± 9.4 Hz to 85.8 ± 8.1 Hz, *p* = 0.94, and from 77.8 ± 12.9 Hz to 78.4 ± 11.6 Hz, *p* = 0.89, respectively) and did not induce a shift in skewness (*p* = 0.56, Figure 2B).

**TABLE 2:** Distribution characteristics of the mean frequencies of acquired point pre and post Atropine administration.

Variables	Pre‐atropine			Post‐atropine			*p* ^b^	
	GPs	Atrium	*p* ^a^	GPs	Atrium	*p* ^a^	GPs	Atrium
Mean, Hz	112.6 ± 13.9	85.9 ± 9.4	**< 0.001**	101.4 ± 13.1	85.8 ± 8.1	**0.001**	**0.02**	0.94
Median, Hz	113.4 ± 18.9	77.8 ± 12.9	**< 0.001**	98.4 ± 16.8	78.4 ± 11.6	**0.002**	**0.02**	0.89
Standard deviation	41.1 ± 5.3	43.8 ± 2.2	0.066	40.3 ± 3.6	43.5 ± 2.9	0.03	0.62	0.75
Curtosis	2.6 ± 0.5	2.5 ± 0.2	0.635	2.5 ± 0.4	2.4 ± 0.3	0.79	0.38	0.42
Skewness	0.03 ± 0.4	0.6 ± 0.2	**< 0.001**	0.3 ± 0.27	0.5 ± 0.2	**0.02**	**0.015**	0.563

*Note:* Data are presented as: Mean ± Standard deviation or *n* (percentage). *p* < 0.05 considered as statistically significant. Statistically significant differences are enhanced in bold.

^a^ *p*‐value of comparison between frequencies of atria and ganglia acquired points pre and post atropine administration.

^b^ *p*‐value of comparison between pre and post atropine administration values of the mean frequency acquired points of the same region (GPs pre vs. GPs post; Atrium pre vs. Atrium post atropine).

## 4 Discussion

Our study systematically evaluated EGM fragmentation and spectral frequency distribution in GPs position and their relation with vagal activity. The main findings can be summarized as follows:

1. GP areas exhibit high signal fragmentation, corresponding to a distinct spectral pattern when compared with the remaining atrial myocardium.
2. Changes in parasympathetic tone induce variations in frequency spectrum and EGM fragmentation exclusively within GP areas, confirming a link between these properties and local neural activity.
3. The spectral difference between GPs and surrounding atrial myocardium persists after vagolysis, suggesting that fixed cellular discontinuity, beyond autonomic tone, may characterize the structural core of GP regions, raising the hypothesis for a possible more precise targeting in the context of cardioneuroablation.

### 4.1 From Spectral Analysis to Fragmentation: Background and Open Questions

Catheter ablation of GPs has been proposed for the treatment of neuro‐mediated syncope. However, targeting GPs through endocardial ablation could be challenging due to the distance between the endocardium and GPs, as well as the difficulty in locating the exact projection of GPs from the endocardial side [5, 8, 10, 11, 12, 14]. In fact, traditional electrophysiological tests and mapping techniques cannot provide direct information regarding the localization of GPs. Previous studies have attempted to identify markers of neuro‐myocardial interface on local EGM [14, 18]. Pachon et al. described a typical “fibrillar” pattern in GPs locations using spectral analysis with fast‐Fourier transform [14]. The authors postulated that the underlying mechanism could be histological discontinuity in GPs areas, where parasympathetic fibers and cardiomyocytes are interposed. The use of spectral analysis to identify ganglionated plexi during ablation procedures is currently limited due to the complexity of the processing required and the difficulty of integrating such analysis into electroanatomic maps generated by modern mapping systems. For this reason, most authors have shifted toward the analysis of electrogram fragmentation (FEGM), which is more easily obtained and reproducible, as a surrogate for spectral analysis in identifying ganglionated plexi [11, 19, 20, 21]. Lellouche et al. described a relationship between EGM fragmentation and parasympathetic response during AF ablation [18] finding that the presence of at least four EGM deflections was the strongest predictor of local vagal response during RF delivery; in addition, in a subgroup of eight patients, they observed that adenosine infusion induced a transformation from normal to fragmented EGM. These findings led the authors to postulate that EGM fragmentation is directly influenced by the local acetylcholine effect. EGM fragmentation and ‘fibrillar’ spectral pattern are likely manifestations of the same underlying phenomenon; however, their correlation has never been systematically investigated and it remains even less clear whether these properties are linked to a functional neural effect or to purely histological characteristics of atrial tissue.

### 4.2 Spectral Signature of GP Regions and Its Selective Modulation by Parasympathetic Tone

In the present study, we sought to revisit spectral analysis for the characterization of the atrial myocardium, under the hypothesis that this methodology is more accurate and tissue‐specific than fragmentation analysis alone: indeed, by avoiding signal filtering, it enables the assessment of all signal components. The main protagonist in vagal modulation of the heart are Atropine, a competitive and reversible antagonist of the muscarinic acetylcholine receptors and Acetylcholine, the primary neurotransmitter of parasympathetic fibers, exerting multiple cellular effect on cardiomyocytes, including hyperpolarization, reduced excitability, slowed conduction, and decreased intercellular coupling [22, 23]. At the dose of 0.04 mg/kg atropine can be safely administered in healthy subjects to block the vagal control of the heart [24]. Our findings demonstrate that GP areas exhibit high signal fragmentation, corresponding to a distinct spectral pattern. Moreover, only in these regions of “fibrillar” myocardium the frequency spectrum is influenced by parasympathetic tone, as evidenced by a clear leftward shift in frequency distribution (increase in skewness) after vagolysis, making it more similar to that of “compact” myocardium. In contrast, no changes in frequency distribution were observed in the rest of the right atrial myocardium following atropine challenge administration. These findings provide in vivo evidence that acetylcholine (ACh) influences atrial EGMs and is not uniformly released across the atrial wall, but rather, is primarily concentrated in GPs regions.

### 4.3 Beyond Autonomic Tone: A Structural‐Functional Basis for Spectral GP Signal Modification

**FIGURE 3:**
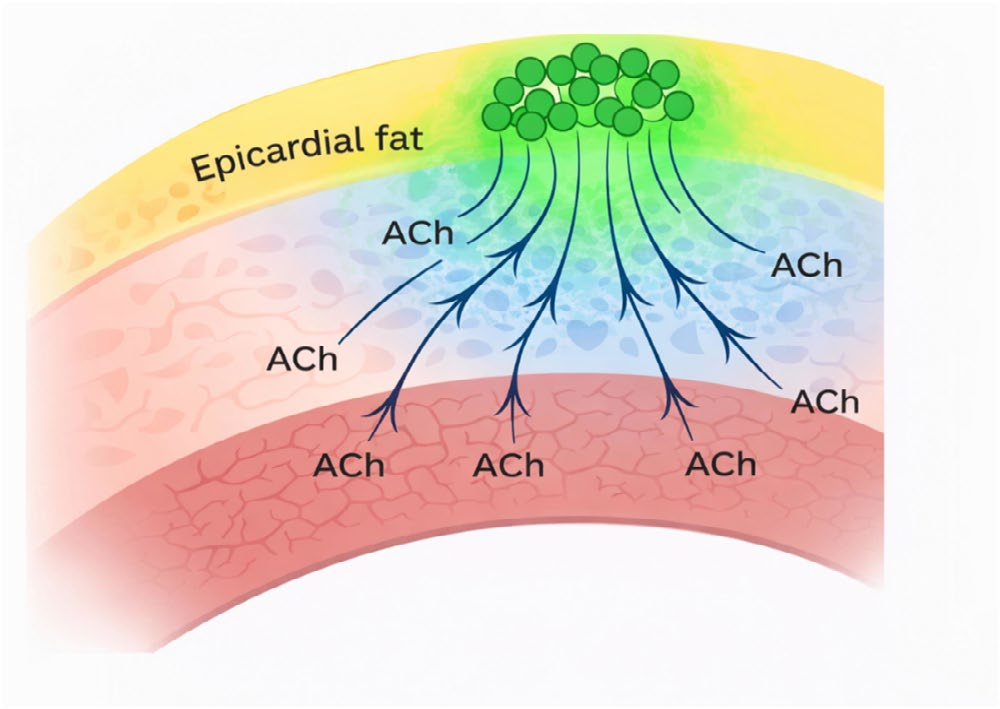
Schematic representation of atrial wall. Transmural section. Neural cell's bodies are contained in the GPs within the epicardial fat layer. The central green area represents the region with highest density of penetrating fibers and histological discontinuity. The surrounding blue area represents the terminal branching zone of neural fibers. Histological architecture is similar to the rest of myocardium however the acetylcholine effect can be observed on local EGM during vagal activity.

Nevertheless, it should be noted that vagolysis changed the spectral profile of GP regions toward that of compact myocardium but did not completely abolish the difference, implying that the signal pattern of GP areas cannot be attributed solely to autonomic tone. In fact, we hypothesize that the mechanism may result from both histological and functional phenomena: the histological architecture is probably the main determinant of the GPs area where the penetrating fibers from the GPs are most concentrated and cellular discontinuity is maximal: this represents a fixed structural property that persists regardless of autonomic tone. On the other hand, in the surrounding of this “central core”, the presence of terminal branching area of parasympathetic fibers and the release of Ach modifies the EGM fragmentation and spectral distribution in a tone‐dependent manner [15] (Figure 3). Our findings confirm that fragmentation is a dynamic property of atrial EGMs, maximal during vagal activity and minimal during vagolysis. The observed relationship between fragmentation and spectral properties suggests that high‐density mapping of the atria provides additional information for the identification of atrial regions corresponding to ganglionated plexi. Although the clinical relevance of these findings in the context of cardioneuroablation requires further validation in dedicated studies, our data suggest that the core of GP regions, where fragmentation and spectral frequencies peak, may represent the most appropriate target for ablation, rather than peripheral areas where the observed signal fragmentation is dependent on vagal tone because mainly mediated by Ach release. This approach, if confirmed in a larger cohort and specifically in dedicated CNA patients, could advise in more targeted ablations, potentially reducing procedural risks and improving long‐term outcomes.

### 4.4 Study Limitations

The main limitation of this study is that the atrial mapping was limited to the right atrium, as left atrial catheterization was not required for scheduled EP procedures. We acknowledge the anatomical asymmetry of the cardiac autonomic nervous system, and in their preferential functional targets [25, 26]. The two atria differ in the number, density, and distribution of epicardial ganglia, with approximately half of all cardiac ganglia concentrated on the posterior and posterolateral surfaces of the left atrium [3, 27]. However, the majority of cholinergic fibers access the human heart through the right atrium [1, 28]. Also if it is therefore reasonable to hypothesize that the electrophysiological phenomena observed in both atria share the same underlying mechanisms, our findings, obtained from the right atrium, should therefore be interpreted within this regional specificity. A further limitation is the absence of an independent, imaging‐based confirmation of GP localization. However, no validated gold‐standard technique currently exists for this purpose, and our regional definition relied on the combination of electrogram fragmentation plus anatomical landmarks, consistent with the most common approach in the fragmentation‐based CNA literature. Another limitation is that, unlike MAP ATP, where the vagal output is pharmacologically blocked by atropine, there is no an equivalent method to induce clear vagal activation during the acquisition of MAP 0: vagal stimulation systems are mostly invasive and poorly tolerated [29]. Nevertheless, a post‐atropine heart rate variation of at least 25% serves as a reliable indicator of vagal tone presence under baseline conditions [17]. Pharmacological autonomic challenge was restricted to parasympathetic blockade with atropine, but GPs contain both parasympathetic and sympathetic elements: the sympathetic contribution to GP electrogram properties therefore remains to be addressed in future studies with a double‐blockade protocol. A potential confounder could be considered the atropine‐induced heart rate increase, which might in principle alterate the spectral composition of atrial EGMs. However, in our case, the spectral changes were confined to GP regions, while the rest of the atrium, exposed to the same heart rate rise, served as an internal control and did show almost any change. The direction of the shift was also opposite to that expected from a rate effect, since faster rates should increase fragmentation and move the spectrum toward higher frequencies, not lower. Finally, all patients stayed in sinus rhythm throughout the protocol, ruling out changes in wavefront orientation as a cause of the observed differences. The aim of the present findings is confined to a potential cardioneuroablation field application and should not be extrapolated to AF ablation, which targets different anatomical regions and addresses a distinct pathophysiological rationale. The relatively small sample size may limit the generalizability of our findings; however, the consistent spectral pattern observed across all patients and the use of high‐density mapping with thousands of data points per map strengthen the reliability of the analysis. In the end, our study was intentionally limited to young and healthy patients with no structural heart disease, in order to have the lowest chance of interfering clinical features; besides, these patients are those with the highest chance to undergo CNA. However, it might be possible that our findings are not directly generalizable to sicker or older patients, as different factors, such as an increased burden of fibrosis, might hamper the effect of vagal activity on the right atrium, both in terms of pattern and amount of fragmentation.

## 5 Conclusions

Frequency spectrum and EGM fragmentation represent closely linked properties that are most evident within ganglionated plexi regions and dynamically vary with parasympathetic tone, underscoring autonomic modulation of local atrial signals. These observations suggest that spectral features may complement electrogram fragmentation in the characterization of atrial regions associated with ganglionated plexi. Moreover, the incomplete reversibility of spectral changes after vagolysis suggests that fixed cellular discontinuity, beyond autonomic tone, may define the structural core of GP area, potentially advising a more precise targeting in the context of cardioneuroablation.

## Aknowledgments

We would like to thank Roberto Formoso and Luca Tropeano for their valuable collaboration.

## Funding

This research did not receive any specific grant from funding agencies in the public, commercial, or not‐for‐profit sectors.

## Conflicts of Interest

The authors declare no conflicts of interest.

## Data Availability

The data that support the findings of this study are available from the corresponding author upon reasonable request.
